# M6A RNA modification: focusing on non-small cell lung cancer progression, therapeutic strategies and challenges

**DOI:** 10.3389/fonc.2025.1622359

**Published:** 2025-07-16

**Authors:** Yuyang Yan, Jiarui Yin, Quan Ding, Yan Lu, Shuhua Gou, Xi Xu, Yulin Li

**Affiliations:** School of Medical and Life Sciences, Chengdu University of Traditional Chinese Medicine, Chengdu, China

**Keywords:** M6A, non-small cell lung cancer, therapeutic strategies, metabolic reprogramming, RNA epigenetic regulation

## Abstract

N6-methyladenosine (m6A) modification is a pivotal mechanism in RNA epigenetics, with profound implications for lung cancer (LC) biology. This review synthesizes current knowledge on m6A’s multifaceted regulatory networks in non-small cell lung cancer (NSCLC), elucidating its roles in tumor proliferation, apoptosis, invasion, and metastasis. We further explore how m6A governs metabolic reprogramming—including glycolysis and ferroptosis—angiogenesis, and tumor microenvironment (TME) remodeling. Additionally, m6A-mediated modification of non-coding RNAs contributes to LC malignancy, underscoring its potential as a diagnostic and prognostic biomarker. These findings also offer novel strategies to overcome therapeutic resistance, a critical challenge in NSCLC treatment. Despite its promise, clinical translation of m6A-targeted interventions faces hurdles, such as the lack of standardized detection methods, the complexity of m6A-associated regulatory networks, and unresolved crosstalk with other RNA modifications. Future research should prioritize multi-omics approaches to resolve these challenges and advance m6A from mechanistic discovery toward clinical application. By addressing these gaps, m6A modulation may emerge as a transformative avenue in precision oncology.

## 1 Introduction

Epigenetic regulation governs the temporal control of gene expression through chemical modifications and chromatin remodeling, achieving heritable modulation without altering the underlying DNA sequence (1). Among these mechanisms, RNA methylation has become a focal point in post-transcriptional regulation research, owing to its reversible and dynamic characteristics (2). As the most prevalent internal modification of eukaryotic mRNA, m6A has driven significant advancements in epigenetic studies (3). Scientists have systematically uncovered its tissue-specific distribution, the functional architecture of its modifying enzyme networks, and its regulatory mechanism in cell fate determination and its diagnostic potential (4, 5). Under physiological conditions, m6A methylation orchestrates the precise regulation of gene expression, maintaining differentiation programs and ensuring the stability of the tissue microenvironment (6, 7). Conversely, in pathological states, disruptions of m6A regulatory networks are closely associated with the etiology and progression of various human diseases (8). Aberrant m6A modifications perturb essential biological processes, including cell cycle regulation, apoptotic pathways, and immune responses, thereby contributing to disease pathogenesis (9, 10).

According to GLOBOCAN 2022 data, LC is the leading cause of cancer-related deaths worldwide, with NSCLC accounting for over 80% of cases. NSCLC poses a significant global health threat due to its high incidence, aggressive nature, and poor prognosis (11). The development of LC is closely linked to aberrant epitranscriptomic regulation (12). Recent studies indicate that RNA methylation, particularly m6A, plays a critical role in LC initiation, metastasis, and treatment resistance (13). Sequencing data reveal dynamic changes in m6A modification levels in NSCLC tissues and circulating tumor RNA, potentially impacting tumor malignancy by regulating oncogene and tumor suppressor gene alternative splicing, stability, and translational efficiency (14).Although initial research has outlined the regulatory network of m6A in LC, a systematic understanding of its precise mechanisms in tumor heterogeneity, microenvironment interactions, and clinical translation remains lacking. Further exploration in this field could not only elucidate the molecular pathogenesis of LC but also offer new epigenetic intervention targets for personalized therapy. For instance, recent studies have shown that compounds like cycloastragenol can modulate the AMPK/ULK1/mTOR signaling pathway to regulate apoptosis and autophagy in NSCLC, highlighting additional layers of epigenetic control relevant to tumor progression and therapeutic resistance (15, 16).

In the broader context of epigenetic regulation, m6A RNA modification does not function in isolation but closely interacts with canonical DNA methylation, particularly 5-methylcytosine (5mC) at CpG islands (17). DNA methylation, as a stable and heritable mark, contributes significantly to gene silencing and tumorigenesis (18). Increasing evidence suggests a regulatory interplay between RNA and DNA epigenetic layers (19). For instance, m6A methylation may influence the transcription of DNA methyltransferases such as DNMT3B, thereby indirectly modulating CpG methylation status (20). Conversely, hypermethylation of promoter regions encoding m6A regulators may attenuate their expression (21). Such bidirectional crosstalk reveals a complex but coordinated epigenetic landscape that governs gene expression programs involved in NSCLC progression and therapeutic resistance.

This paper offers a comprehensive overview of the molecular mechanisms governing m6A modification and recent innovations in detection methodologies. It focuses specifically on the multifaceted roles of m6A in NSCLC progression, covering tumor growth, metastasis, metabolic reprogramming (such as glycolysis and ferroptosis), and TME interactions. Additionally, we discuss the diagnostic and therapeutic potential of m6A-related factors—namely the “Writers,” “Erasers,” and “Readers”—in biomarker development, overcoming therapeutic resistance, and prognostic evaluation. Through an in-depth literature review, we aim to propose novel insights and perspectives for advancing early detection and personalized treatment strategies for NSCLC.

## 2 m6A modification: architecture and functional dynamics

**Figure 1:**
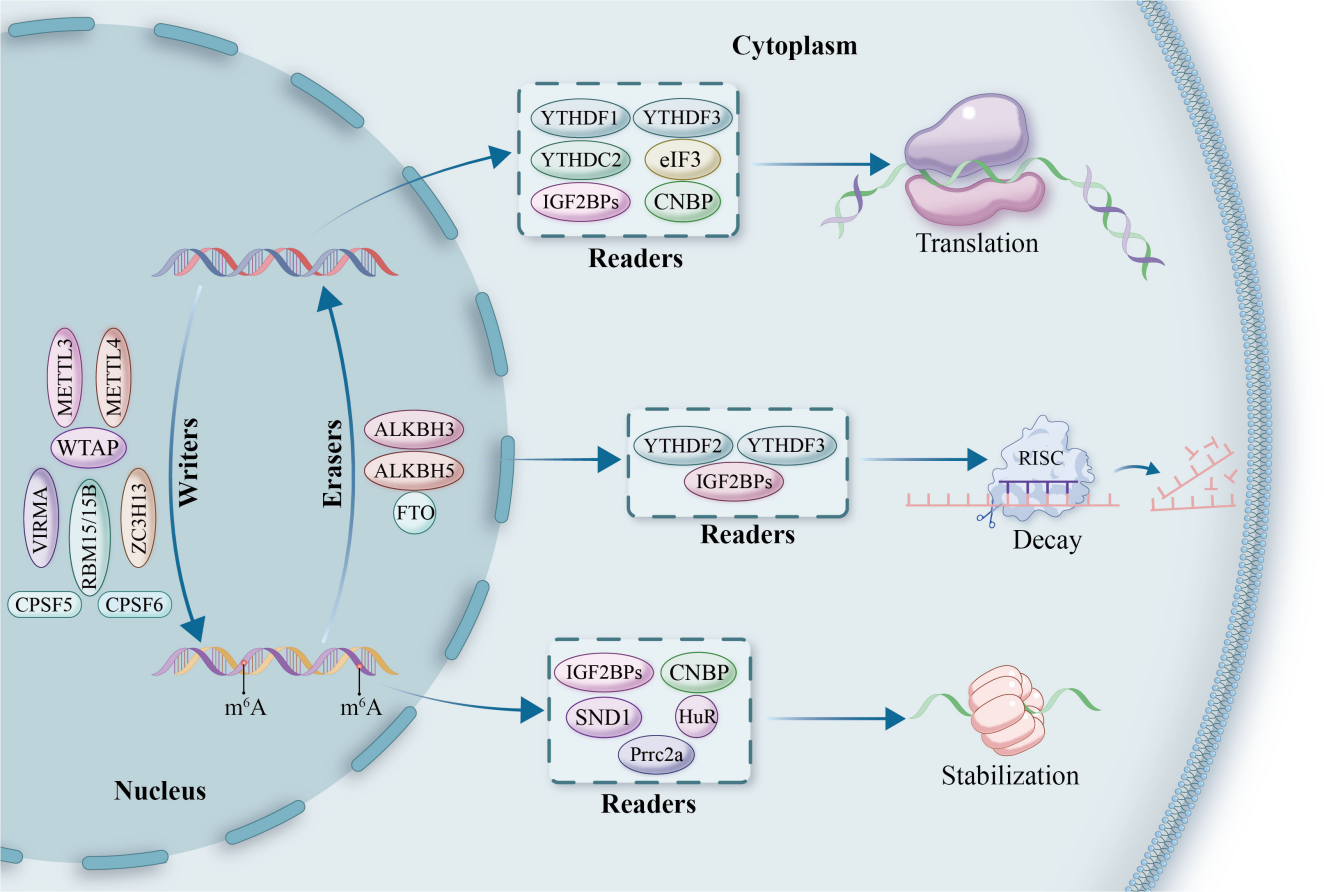
The mechanistic framework of m6A modification in RNA regulation. This diagram illustrates the roles of m6A-modifying proteins, classified into ‘writers,’ ‘erasers,’ and ‘readers.’ Writers, such as METTL3, METTL4, VIRMA, ZC3H13, WTAP, and RBM15/15B, are responsible for adding methyl groups to RNA in the nucleus. Erasers, including FTO, ALKBH5, and ALKBH3, remove these modifications, enabling dynamic reversibility in gene regulation. Readers, such as YTHDF1, YTHDF2, YTHDF3, YTHDC2, and IGF2BPs, bind to methylated RNA and influence its downstream fate, orchestrating processes of translation, decay, and stabilization. Additional factors such as HuR, eIF3, SND1, and Prrc2a play supporting roles in translation regulation, while CPSF5 and CPSF6 are involved in RNA processing and nuclear-cytoplasmic transport. This comprehensive overview highlights the intricate and multifaceted nature of m6A-mediated gene expression control.

m6A modification is a central mechanism of epigenetic regulation in eukaryotic RNA, participating in the precise regulation of gene expression through dynamic and reversible methylation (22) (**Figure 1**). This modification is widely present in various RNA molecules, exhibiting a highly conserved RRACH (R=G/A; H=A/C/U) sequence preference in mammalian mRNA, predominantly enriched in coding sequences (CDS) and 3’ untranslated regions (3’UTR), with modification sites appearing approximately once every 700-800 nucleotides (23).High-throughput sequencing studies have revealed that over 12,000 genes in the human genome carry m6A modifications, indicating its widespread involvement in transcriptomic regulation (1, 24). The dynamic regulation of m6A modification is maintained by three classes of functional proteins: methyltransferases (Writers), demethylases (Erasers), and methylation readers (Readers) (25) (**Table 1**).

**Table 1:** The roles of m6A regulators in RNA metabolism.

Type	Proteins	Location	Functions	Ref
Writers	METTL3	Cytoplasm and Nucleus	Regulating RNA stability, translation efficiency, and splicing through catalyzing m6A modification.	(26)
	METTL4	Nucleus	Functions as a methyltransferase implicated in U2 snRNA modification and potentially involved in chromatin organization.	(27)
	METTL14	Nucleus	Assisting the conformational stability of METTL3 and enhancing substrate recognition specificity	(28)
	METTL16	Cytoplasm and Nucleus	Regulating the balance of SAM metabolism and RNA translation stability through m6A modification.	(29)
	RBM15	Nucleus	Specifically recruiting the m6A methylation complex to regulate RNA modification, stability, and nuclear transport.	(30)
	WTAP	Nucleus	Can regulate the degradation rate and translation efficiency of RNA.	(31)
	VIRMA	Nucleus	Responsible for guiding the m6A modification complex to specific regions of RNA, thereby regulating the localization and function of target RNA.	(32)
	ZC3H13	Nucleus	Maintaining the nuclear function of the m6A methylation machinery by anchoring the WTAP-RBM15 complex.	(33)
Erasers	FTO (ALKBH9)	Nucleus	Dynamically reversing m6A modification by oxidizing m6A to hm6A and f6A, thereby regulating RNA stability.	(34)
	ALKBH5	Nucleus	Directly removing the m6A methyl group, thereby affecting RNA splicing and nucleocytoplasmic transport.	(35)
	ALKBH3	Cytoplasm and Nucleus	Participating in tRNA demethylation, thereby enhancing translation efficiency and DNA repair.	(36)
Readers	YTHDF1	Cytoplasm	Binding to eIF3 to promote the translation initiation of m6A-modified mRNA.	(37)
	YTHDF2	Cytoplasm	Recruiting the CCR4-NOT complex to direct m6A-mRNA to P-bodies for degradation.	(38)
	YTHDF3	Cytoplasm	Synergizing with YTHDF1/2 to bidirectionally regulate mRNA translation and degradation.	(39)
	YTHDC1	Nucleus	Regulating mRNA splicing and nuclear export through the SRSF3 and TREX complexes.	(36)
	YTHDC2	Cytoplasm	Binding to XRN1 to promote the degradation of m6A-mRNA while enhancing translation.	(40)
	IGF2BP1	Cytoplasm	Stabilizing target mRNA and promoting its translation while resisting degradation.	(41)
	IGF2BP2	Cytoplasm	Stabilizing target mRNA and promoting its translation while resisting degradation.	(42)
	IGF2BP3	Cytoplasm	Stabilizing target mRNA and promoting its translation while resisting degradation.	(43)
	HNRNPC	Nucleus	Regulating alternative splicing and affecting RNA processing by binding to m6A-modified pre-mRNA.	(44)
	HNRNPG	Nucleus	Regulating alternative splicing and affecting RNA processing by binding to m6A-modified pre-mRNA.	(45)
	HNRNPA2B1	Nucleus	Recognizing m6A modification to regulate pre-mRNA splicing and abundance.	(46)
	eIF3	Cytoplasm	Regulating the translation efficiency of m6A-mRNA by assembling the translation initiation complex.	(47)

m6A, N6-methyladenosine; SAM, S-adenosylmethionine; hm6A, N6hydroxymethyladenosine; SAM, S-adenosylmethionine; CCR4-NOT, CCR4-NOT complex; P-bodies, Processing bodies; SRSF3, Serine/arginine-rich splicing factor 3; TREX, Transcription/export complex; XRN1, 5’-3’ exoribonuclease 1.

These regulators critically influence cell fate by modulating RNA splicing, nucleo-cytoplasmic transport, translation efficiency, and RNA stability (48). Importantly, aberrant m6A modifications have been implicated in the progression of numerous malignancies. In LC specifically, dysregulated m6A signaling contributes to tumorigenesis and therapeutic resistance by facilitating oncogene activation, silencing tumor suppressor genes, promoting immune evasion, and driving metabolic reprogramming (13, 49).

### 2.1 Writers: catalysts of m6A installation

The establishment of m6A modification is mediated by a multi-protein methyltransferase complex (MTC), with METTL3 serving as the core catalytic subunit (50). METTL3 catalyzes the transfer of a methyl group from SAM (S-adenosylmethionine) to the N6 position of adenosine within RNA molecules (51). Although METTL14 lacks intrinsic catalytic activity, it enhances methylation efficiency by stabilizing METTL3’s structural conformation and facilitating RNA substrate recognition (52). The METTL3/METTL14 heterodimer selectively binds to conserved GGACU motifs, thereby conferring sequence specificity to m6A modifications. In addition to the core catalytic units, MTC includes several regulatory subunits: WTAP acts as a scaffold protein and aids the assembly of the complex by interacting with METTL3-METTL14, thereby engaging in RNA targeting and splicing regulation (53);VIRMA directs regional methylation deposition of MTC at the 3’UTR and near stop codons (54).

ZC3H13 links RBM15 to WTAP, maintaining the complex’s nuclear localization (55);RBM15 and its homolog RBM15B bind specific RNA structures, further expanding the substrate recognition range of MTC (56). Additionally, METTL16, acting independently of the canonical MTC, regulates the m6A modification patterns of specific non-coding RNAs (57). These components collaborate intricately to ensure the spatiotemporal specificity of m6A modifications across the transcriptome.

### 2.2 Erasers: reversing the m6A marks

RNA demethylases, known as “Erasers,” provide the molecular basis for the reversibility of m6A modifications, intricately regulating the dynamic balance of methylation. To date, identified m6A demethylases belong to the α-ketoglutarate-dependent dioxygenase family, utilizing 2-oxoglutarate (2OG) and divalent iron ions (Fe(II)) as essential cofactors (58, 59). FTO was the first demethylase discovered, functioning through a two-step oxidative demethylation process: first, oxidizing m6A to an unstable intermediate, N6-hydroxymethyladenosine (hm6A), followed by further oxidation to N6-formyladenosine (f6A), which spontaneously hydrolyzes into adenosine (A) in aqueous environments (60, 61). ALKBH5 (AlkB homolog 5) employs a different catalytic strategy as this nuclear-localized protein directly removes the methyl group to demethylate m6A (62). Notably, ALKBH3, another family member, primarily participates in the demethylation of tRNA, broadening the functional scope of this enzyme family (63). These three enzymes exhibit distinct differences in spatial distribution and substrate preference, collectively forming a complex and precise network for m6A demethylation regulation.

### 2.3 Readers: decoding the m6A signals

The biological effects of m6A modifications are primarily mediated by specific recognition proteins known as “Readers,” which include the YTH domain family, the IGF2BP family, the heterogeneous nuclear ribonucleoprotein family, and the eukaryotic initiation factor 3 (eIF3) (64). The YTH domain, a highly conserved m6A recognition module, determines the specific binding ability of these proteins to m6A sites in RNA.

YTHDF2 selectively binds m6A-modified RNAs with its C-terminal YTH domain. Its N-terminus binds the SH domain of CNOT1 subunit of the CCR4-NOT complex to recruit the deadenylation complex (65). This action guides m6A-tagged mRNAs to processing bodies (P-bodies) for poly(A) tail shortening and subsequent mRNA degradation (66). YTHDF1 also binds m6A-tagged mRNA using its YTH domain and interacts with the translation initiation factor eIF3, enhancing initiation of translation (37). Notably, YTHDF1 activity depends on a mechanism of mRNA circularization through eIF4G, largely enhancing translational efficacy (67). Meanwhile, YTHDF3 collaborates with YTHDF1 and YTHDF2 for coordination of m6A-derived transcription with stability regulation of m6A-modified mRNAs in the cytoplasm (68). Acting as a key m6A nuclear reader, YTHDC1 controls RNA nucleocytoplasmic transport by binding with serine/arginine-rich splicing factor 3 (SRSF3), nuclear export factor 1 (NXF1), as well as the TREX complex, thereby controlling mRNA splicing and export (69). YTHDC2 possesses distinct dual functions: its 3’/5’ RNA helicase activity promotes m6A-modified mRNA degradation, while binding with 5′→3′ exoribonuclease 1 (XRN1) promotes mRNA stability under some circumstances, making it possible to control the exact regulation of target mRNA cis/translational efficacy as well as its abundance with precision (70, 71).

Additionally, m6A modifications have a profound effect on RNA secondary structure. HNRNPC and HNRNPG, heterogeneous nuclear ribonucleoproteins, recognize such structural changes, controlling mRNA levels and alternative splicing through a mechanism referred to as the “m6A switch” (45). During precursor mRNA splicing, these recognition proteins act coordinately to modulate several important attributes such as transcript stability, splicing, nucleocytoplasmic transport, and translation efficacy. METTL3-dependent recognition of m6A core site RGAC is specifically mediated through HNRNPA2B1, with regulation of alternative splicing of RNA, as well as precursor miRNAs maturation (72). IGF2BP family proteins also recognize target mRNA m6A-dependently and recruit multiple RNA stability factors, establishing a pivotal mechanism of mRNA stability control as well as for regulating translation efficacy (73, 74). These studies systematically demonstrate the complexity as well as precision of m6A recognition protein network regulating RNA metabolism, augmenting insights into epitranscriptomic control mechanisms at a molecular level.

## 3 Emerging technologies for m6A detection

Following the initiation of RNA modification research in the 1970s, the m6A research has witnessed a huge technological progress. 2012 was a turning point with the advent of new RNA immunoprecipitation accompanied with next-generation sequencing (NGS) technologies, which succeeded in bringing revolutionary progress to m6A research (75). Initial technical platforms were unable to accurately identify m6A positions due to the limitation of detection, a problem solved only with the full utilization of high-throughput sequencing technologies (76). In this technological path, MeRIP-seq/m6A-seq enriched m6A-methylated mRNA fragments for use with NGS, achieving methylation peak detection at a resolution of 100-200 nucleotides for the first time, yet it still could not precisely locate individual m6A modification sites across the complete transcriptome (77). The PA-m6A-seq technique introduced a 4-thiouridine (4SU) enhanced crosslinking strategy, improving detection resolution to about 30 nucleotides, although its application was limited by localized detection capacities around 4SU labeling sites (78).

To overcome these technical limitations, researchers developed the miCLIP technology for m6A single-nucleotide resolution crosslinking immunoprecipitation. This innovative method, by optimizing crosslinking conditions and antibody characteristics, successfully identified m6A sites at single-nucleotide resolution, marking a new era of precise localization in epitranscriptomic research (79, 80). In recent years, m6A modification detection technologies have achieved unprecedented breakthroughs, enabling true single-nucleotide resolution analysis. The m6A-REF-seq technique uses the RNA endonuclease MazF in conjunction with specific antibody enrichment, allowing precise localization and quantification of m6A sites across the entire transcriptome, significantly enhancing detection reliability and coverage (81). More innovative is the DART-seq technology, which abandons traditional antibody-based methods and cleverly employs cytidine deaminase (APOBEC1) fused with the YTH domain to convert cytidine (C) adjacent to m6A into uridine (U), enabling highly sensitive, antibody-free detection by analyzing C-to-U editing events (82).

Concurrently, the m6A-label-seq technique, based on metabolic labeling strategies, showcases unique advantages. It converts m6A into N6-allyladenosine (a6A), which, under iodination, forms N1,N6-cyclized adenosine (cycA), ultimately inducing A→C/T/G mutations during reverse transcription, accurately identifying m6A modification sites at a single-nucleotide level (83). Another key breakthrough is from the m6A-SEAL technology, which utilizes the oxidative properties of the FTO demethylase and DTT-mediated chemical reactions to specifically label and biotinylate m6A sites, combining with high-throughput sequencing to simultaneously enhance detection sensitivity and coverage (84).

The iterative advancement of m6A detection technologies has developed into a multifaceted and complementary system. m6A-seq2, by integrating multiplex sample labeling and barcode tracking, significantly boosts experimental throughput by several orders of magnitude, with its optimized statistical model markedly improving quantitative accuracy in cross-sample comparative studies (85). miCLIP2 technology introduces dual innovations on its original foundation: microfluidic enhancements in the experimental process, combined with the m6A Boost machine learning algorithm, elevate detection sensitivity to sub-microgram RNA levels while systematically optimizing the false-positive rate (86). m6ALAIC-seq ingeniously introduces UV crosslinking to stabilize RNA-protein complexes, paired with high-specificity antibody enrichment, achieving modification localization at single-nucleotide resolution (87).

In the realm of targeted validation, SELECT, MeRIP-qPCR, and MazF-qPCR technologies complement each other, providing precise quantitative tools for dynamic m6A monitoring at the single-gene level (88). The SCARLET method, combining radioactive labeling with thin-layer chromatography (TLC), has established an antibody-independent RNA modification localization system, suitable for precise analysis of various RNA molecules (89). Furthermore, mass spectrometry (MS), dot blotting, and colorimetry continue to serve as crucial support in fundamental research, providing reliable data for the global assessment of m6A levels (90, 91).

Despite substantial technological advances, challenges remain for the clinical application of m6A detection platforms. These include the high cost of equipment and reagents, limited throughput for large-scale clinical samples, and the need for highly specialized technical expertise (92). Additionally, the lack of standardized protocols across platforms complicates result reproducibility and data integration. Future work should prioritize cost-effective, scalable, and user-friendly approaches to facilitate translational adoption in clinical oncology.

## 4 Functional roles of m6A in the normal pulmonary microenvironment

### 4.1 Regulation of pulmonary immune cell function by m6A

m6A RNA methylation plays an indispensable role in maintaining pulmonary immune microenvironment homeostasis. As a reversible epigenetic mark, m6A modulates immune cell function by altering the stability, splicing, and translation of immune-related transcripts (93). In alveolar macrophages (AMs), m6A modifications orchestrate the balance between pro-inflammatory (M1) and anti-inflammatory (M2) polarization by influencing the expression dynamics of key immune regulators. For instance, Cao et al. proved that YTHDF1 facilitates the enhanced translation of m6A-methylated GBP4 mRNA, thus favoring M1 macrophage polarization as well as accelerating acute pulmonary inflammation (94). Under homeostatic conditions, m6A methylation mediated by METTL3 inhibits excessive activation of macrophages by stabilizing negative regulators of inflammation including suppressor of cytokine signaling 1 (SOCS1) as well as suppressor of cytokine signaling 3 (SOCS3) to inhibit inflammatory injury of the lungs.

Moreover, the m6A-bound protein LRPPRC negatively controls macrophage inflammatory responses, with its deficiency leading to the increased expression of TNF-α as well as IL-6, indicating its role in maximizing macrophage immune functions (95). Dendritic cell antigen presentation is similarly regulated by m6A methylation. Chen et al. (2025) indicated that dendritic cell (DC) with increased m6A content exhibit increased expression of MHC-II as well as co-stimulatory molecules (CD80/CD86), hence augmenting T cell activation (96). Conversely, viral infection leads to global m6A demethylation, which inhibits antigen presentation of DC as well as immune escape of the pathogen (97).

### 4.2 Dynamic balance of immune responses mediated by m6A

m6A RNA methylation serves as a central regulator in maintaining pulmonary immune homeostasis by coordinating a precise balance between innate and adaptive immunity through multi-layered, dynamically reversible modification mechanisms. As a critical organ in constant contact with the external environment, the respiratory system faces the dual challenge of effectively clearing pathogens while avoiding excessive inflammatory responses. Recent studies have revealed that this balance relies, in part, on the subtle regulation of immune key molecule expression by m6A modifications (98). During the pathogen recognition phase, the mRNA of pattern recognition receptors generally exhibits high levels of m6A modification. This modification maintains low-level expression under baseline conditions while allowing rapid transcriptional activation during infection, ensuring timely immune responses without triggering autoimmune damage (99).

Notably, various respiratory viruses (such as SARS-CoV-2 and influenza virus) have evolved the ability to disrupt the host’s m6A modification system by reducing overall m6A levels or selectively removing the m6A markers of antiviral genes (such as those in the type I interferon pathway) to achieve immune evasion (97). Host cells employ a “modification antagonism” strategy, where METTL3-mediated methylation of viral RNA promotes its degradation, while enhancing the m6A modification of key effector molecules like IRF7 and MAVS to increase their translation efficiency (98, 100). At the adaptive immunity level, m6A modifications maintain immune balance by differentially regulating the differentiation and function of T cell subsets: in effector T cells, YTHDF1 promotes the translation of effector molecules such as IFN-γ, while in Treg cells, METTL3 stabilizes FoxP3 expression. This dual regulation ensures precise control over the intensity and duration of immune responses (101, 102).

Additionally, emerging evidence suggests that m6A also participates in the epigenetic regulation of immune checkpoint molecules. Under physiological conditions, YTHDF2-mediated degradation of PD-1 mRNA suppresses autoimmune responses; dysregulation of this mechanism may lead to a breach of immune tolerance or T cell exhaustion (103). Particularly noteworthy is the finding that the gut microbiota remotely regulate m6A modification patterns in pulmonary immune cells via the “gut-lung axis.” Bile acid metabolism-dependent epitranscriptomic remodeling can alter the expression profiles of hundreds of immune-related genes, offering new insights into the systemic regulation of mucosal immunity (104).

### 4.3 Multidimensional integration of m6A in pulmonary homeostasis

From a systems biology perspective, m6A modifications form a multidimensional regulatory network that integrates microenvironmental signals and coordinates the physiological functions of lung tissue. Multi-omics analyses have elucidated the essential role of m6A modification in maintaining the lung’s three critical functional systems: physical barriers, oxidative stress defense, and immune surveillance (105). m6A RNA methylation governs epithelial barrier integrity primarily through post-transcriptional regulation of key genes. For example, m6A modifications stabilize transcripts encoding tight junction proteins such as claudin-2, thereby enhancing barrier function (106). In contrast, loss of the methyltransferase METTL14 leads to aberrant activation of the TNF pathway and pro-apoptotic genes, ultimately disrupting epithelial integrity (107). Beyond junctional proteins, m6A modifications also modulate the expression of secretory factors via long non-coding RNAs (lncRNAs), such as SPRY4-IT1, potentially altering the physicochemical properties of the mucus layer (108).

At the metabolic level, m6A-mediated reprogramming connects immune cell function to the nutritional status of the microenvironment. Selective modification of mRNAs encoding rate-limiting enzymes, such as HK2 in glycolysis and IDH2 in the tricarboxylic acid cycle, enables the metabolic shift critical for M1/M2 macrophage polarization (109, 110). Recent groundbreaking research has discovered that m6A-modified RNA can transmit regulatory signals between different cell types in the lung via extracellular vesicles. m6A-labeled RNA from fibroblasts can influence epithelial barrier function and macrophage activation state. This intercellular “epigenetic communication” might be a crucial coordination mechanism for tissue damage repair (111).

## 5 m6A in NSCLC progression

**Figure 2:**
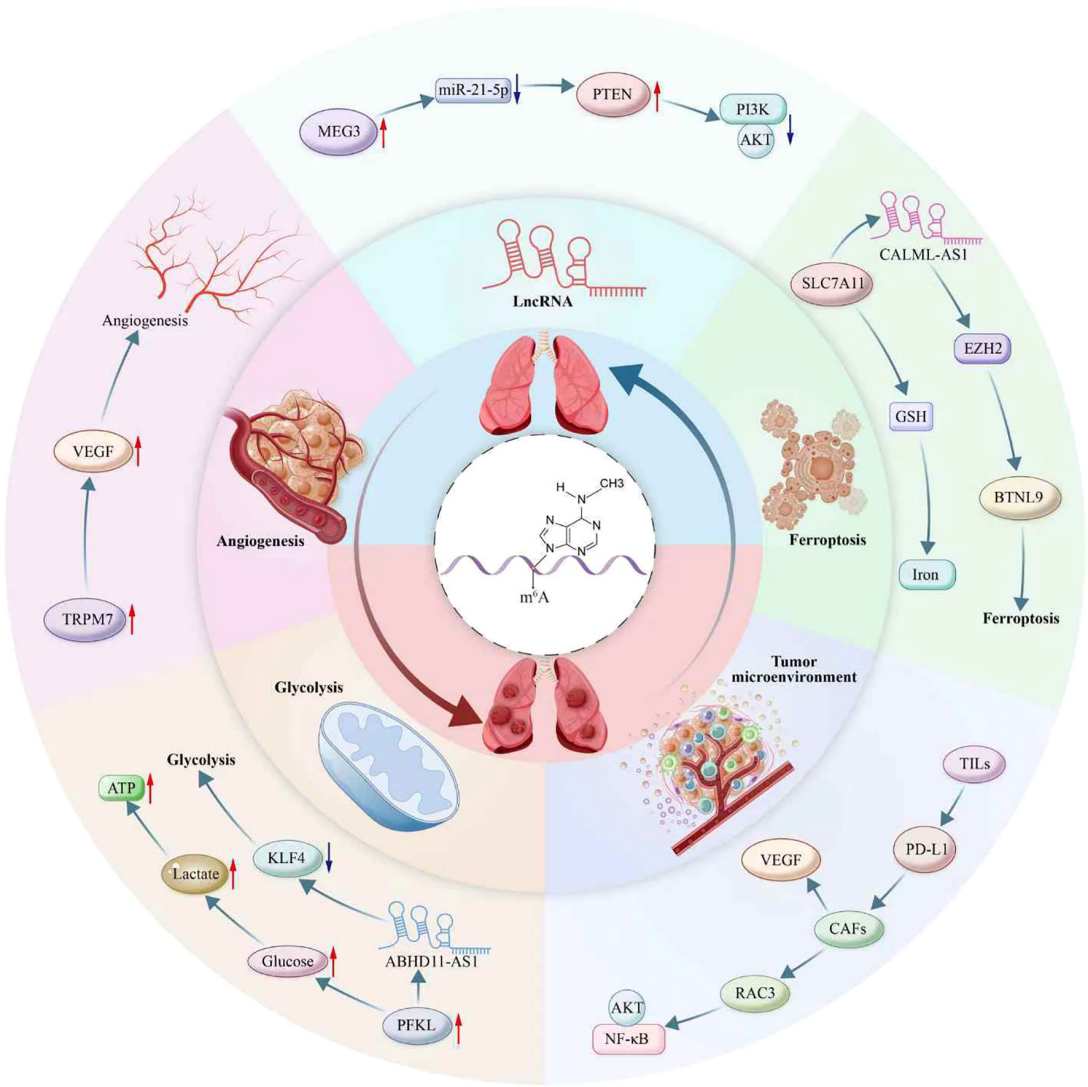
Multifaceted roles of m6A modification in non-small cell lung cancer (NSCLC) pathogenesis. This diagram illustrates m6A’s involvement in diverse biological processes contributing to NSCLC progression, including ferroptosis, tumor microenvironment modulation, lncRNA interaction, angiogenesis, and glycolysis. Key molecular interactions highlighted include ABHD11-AS1 and CALML-AS1 in the regulation of lncRNA; PTEN/AKT/PI3K pathways in cellular survival signaling; and the influence of metabolites like glucose, lactate, and ATP in glycolytic reprogramming. The diagram also depicts the role of m6A in modulating immune cell infiltration and immune checkpoint pathways via TILs and PD-L1, and its impact on angiogenesis through factors like VEGF and RAC3. Additionally portrayed are m6A’s interactions with non-coding RNAs, exemplified by MEG3, miR-21-5p, and their downstream targets like TRPM7, PFKL, and KLF4. Furthermore, m6A-mediated regulation affects the tumor microenvironment, involving players such as CAFs, SLC7A11, GSH, and EZH2, contributing to processes like iron metabolism and activation of signaling pathways including NF-κB. This intricate network underscores the critical role of m6A as an epigenetic regulator in NSCLC.

As a pivotal epigenetic regulator in NSCLC, m6A modification drives tumor progression through multiple dimensions (**Figure 2**). It finely balances proliferation and apoptosis by modulating metabolic pathways, such as glycolysis and ferroptosis, while profoundly influencing angiogenesis and remodeling of the TME. Notably, the m6A-dependent lncRNA regulatory network provides a novel molecular basis for tumor invasion and metastasis, highlighting this RNA modification as a pivotal element in the metastatic cascade of NSCLC (**Table 2**).

**Table 2:** The potential mechanisms and targets of m6A regulators in non-small cell lung cancer (NSCLC).

Classification	m6A Type	Expression	Targeting	Function	Ref
Writers	METTL3	↑	RIG-I	Inhibits the RIG-I-MAVS innate immune pathway, promoting the progression and metastasis of NSCLC, serving as a poor prognostic marker.	(112)
		↑	circIGF2BP3, PD-L1	Elevated circIGF2BP3 expression is associated with lymph node metastasis and advanced tumor stages, suppresses CD8+ T-cell infiltration, impacting prognosis.	(113)
		↑	LINC02418	Involved in the degradation of LINC02418 via YTHDF2, inhibiting METTL3 can upregulate LINC02418 expression, decrease PD-L1 levels, enhancing immunotherapy efficacy.	(114)
		↑	miR-196a, GAS7	Enhances miR-196a stability, inhibits GAS7 expression, reduces immune cell infiltration, facilitates tumor immune evasion, affecting the tumor microenvironment.	(115)
		↑	SLC7A5	CAFs deliver METTL3 to NSCLC cells, upregulating SLC7A5 expression, modulating tumor cell behavior.	(116)
		↑	TRPM7, VEGFA	Stabilizes TRPM7 mRNA, increases VEGFA expression, promotes tumor angiogenesis.	(117)
		↑	PFKL	Stabilizes PFKL mRNA via circDHTKD1 - IGF2BP2 complexes, promoting glycolysis.	(118)
		↑	FSP1	Augmentation of m6A modifications may decrease FSP1 expression, promoting ferroptosis.	(119)
		↑	MALAT1, YAP	Enhances MALAT1 stability, upregulates YAP expression through the MALAT1 - miR-1914-3p - YAP axis, facilitating NSCLC invasion and metastasis.	(120)
	METTL14	↓	LINC02747	Reduces LINC02747 expression, inhibits PI3K/Akt signaling pathway, limits cell proliferation and migration, potentially promoting apoptosis.	(121)
		↓	SLC3A2	Decreases SLC3A2 expression, heightening sensitivity to ferroptosis inducers.	(122)
	KIAA1429	↑	MAP3K2	Modulates MAP3K2 expression, activates JNK/MAPK signaling pathway, leading to gefitinib resistance.	(123)
	VIRMA	↑	DAPK3	Reduces DAPK3 mRNA stability through m6A modifications, inhibiting its expression, exerting carcinogenic effects.	(124 )
	RBM15	↑	CBR3-AS1	Regulates CBR3-AS1 expression, recruits MDSCs, facilitating radiotherapy resistance and tumor invasion & metastasis.	(125)
	WTAP	↑	ERK	Serves as a critical component of the m6A methyltransferase complex, involved in m6A modification processes.	(124)
	ZC3H13	↑	RBM15, WTAP	Links RBM15 and WTAP, maintaining the nuclear localization of the complex.	(103)
	RBM15B	↑	NXF1	Associates with specific RNA structures alongside RBM15, further expanding the substrate recognition scope of the MTC.	(126)
	METTL16	↑	specific non-coding RNAs	Operates independently of typical MTCs, modulating m6A modification patterns of specific non-coding RNAs.	(127)
Erasers	ALKBH5	↑	SLC7A11	Decreases SLC7A11 expression through demethylation, promoting ferroptosis, inhibiting tumor invasion & metastasis.	(128)
		↑	CALML3-AS1	Demethylates CALML3-AS1, promotes NSCLC cell proliferation, recruits EZH2 to suppress BTNL9 expression, advancing invasion & metastasis.	(129)
		↑	FEZF1-AS1, ITGA11	Regulates FEZF1-AS1 expression, upregulates ITGA11 via the miR-516b-5p axis, enhancing NSCLC cell invasion & metastasis.	(130)
		↑	circEML4	Modulates ALKBH5 activity or blocks its interaction with circEML4, inhibiting JAK-STAT signaling pathway activation, thereby curbing NSCLC progression.	(131)
Readers	IGF2BP2	↑	TRPM7, VEGFA	Stabilizes TRPM7 mRNA, increases VEGFA expression, promotes tumor angiogenesis.	(42)
		↑	circDHTKD1	Enhances binding capacity to PFKL mRNA, stabilizes mRNA, promoting glycolysis.	(118)
		↑	FSP1	May affect ferroptosis by maintaining FSP1 mRNA stability.	(132)
		↑	MALAT1, YAP	Potentially regulates YAP expression by affecting MALAT1 stability, impacting NSCLC invasion & metastasis.	(133)
	IGF2BP3	↑	—	Acts as an m6A reader protein, maintains anti-ferroptosis factor mRNA stability, inhibits ferroptosis, enhancing tumor cell survival & invasive capacity.	(134)
	YTHDF1	↑	GBP4	Recognizes m6A-modified GBP4 mRNA, enhances its translation, promotes M1 macrophage polarization, exacerbating inflammatory response.	(94)
		↑	EIF3C	Promotes EIF3C translation, affecting cell cycle progression.	(37)
	YTHDC1	↓	FSP1	May influence ferroptosis by regulating FSP1 mRNA nucleo-cytoplasmic transport.	(135)
	YTHDC2	↑	CALML3-AS1	Identifies m6A modifications on CALML3-AS1, promotes its degradation, suppressing NSCLC cell proliferation.	(40)
	HNRNPA2B1	↑	lncRNA MEG3	Regulates the stability of lncRNA MEG3, consequently affecting the miR-21-5p/PTEN axis, fostering NSCLC pathogenesis and progression.	(136)
	HNRNPC	↑	PD-L1	Associated with an immunosuppressive microenvironment, potentially promoting tumor progression through PD-L1 expression regulation and affecting immune cell infiltration.	(137)
		↓	CD8+ T Cell	Recognizes m6A-modified PFKL mRNA, may impact its stability or translation efficiency, thereby influencing glycolysis.	(138)

m6A, N6-methyladenosine; NSCLC, Non-Small Cell Lung Cancer; METTL3, Methyltransferase-like 3; circIGF2BP3, Circular RNA Insulin-like Growth Factor 2 mRNA Binding Protein 3; PD-L1, Programmed Death-Ligand 1; CD8+ T-cell, Cluster of Differentiation 8 Positive T-cell; YTHDF2, YTH N6-methyladenosine RNA Binding Protein 2; miR, MicroRNA; GAS7, Growth Arrest-Specific 7; SLC7A5, Solute Carrier Family 7 Member 5; CAFs, Cancer-Associated Fibroblasts; TRPM7, Transient Receptor Potential Melastatin 7;VEGFA, Vascular Endothelial Growth Factor A; PFKL, Phosphofructokinase, Liver Type; FSP1, Ferroptosis Suppressor Protein 1; MALAT1, Metastasis Associated Lung Adenocarcinoma Transcript 1; YAP, Yes-Associated Protein; LINC02418, Long Intergenic Non-Protein Coding RNA 2418; PI3K/Akt, Phosphoinositide 3-Kinase/Protein Kinase B; SLC3A2, Solute Carrier Family 3 Member 2; KIAA1429, KIAA1429 Gene Product; MAP3K2, Mitogen-Activated Protein Kinase Kinase Kinase 2; JNK/MAPK, c-Jun N-terminal Kinase/Mitogen-Activated Protein Kinase; VIRMA, Virilizer-like Protein; DAPK3, Death-associated Protein Kinase 3; RBM15, RNA-Binding Motif Protein 15; CBR3-AS1, Carbonyl Reductase 3, Antisense RNA 1; MDSCs, Myeloid-Derived Suppressor Cells; WTAP, Wilms’ Tumor 1 Associated Protein; ERK, Extracellular Signal-Regulated Kinase; ZC3H13, Zinc Finger CCCH-Type Containing Protein 13; RBM15B, RNA-Binding Motif Protein 15B; NXF1, Nuclear RNA Export Factor 1; MTC, Methyltransferase Complex; METTL16, Methyltransferase-like 16; ALKBH5, AlkB Homolog 5; SLC7A11, Solute Carrier Family 7 Member 11; CALML3-AS1, Calmodulin-like 3, Antisense RNA 1; EZH2, Enhancer of Zeste Homolog 2; BTNL9, Butyrophilin-like Protein 9; FEZF1-AS1, FEZF1 Antisense RNA 1; ITGA11, Integrin Subunit Alpha 11; circEML4, Circular Echinoderm Microtubule-Associated Protein-like 4; JAK-STAT, Janus Kinase-Signal Transducer and Activator of Transcription; IGF2BP2, Insulin-like Growth Factor 2 mRNA Binding Protein 2; GBP4, Guanylate Binding Protein 4; M1 Macrophage, Type 1 Macrophage; EIF3C, Eukaryotic Translation Initiation Factor 3 Subunit C; YTHDC1, YTH Domain Containing Protein 1; YTHDC2, YTH Domain Containing Protein 2; HNRNPA2B1, Heterogeneous Nuclear Ribonucleoprotein A2/B1; lncRNA MEG3, Long Non-Coding RNA Maternally Expressed 3; miR-21-5p, MicroRNA 21-5p; PTEN, Phosphatase and Tensin Homolog; HNRNPC, Heterogeneous Nuclear Ribonucleoprotein C; circDHTKD1, Circular Dihydroketoacyl-CoA Synthase 1.

### 5.1 Regulation of proliferation and apoptosis by m6A in NSCLC

#### 5.1.1 TME interactions

In NSCLC, m6A-mediated RNA modifications intricately reshape the TME by regulating immune cell infiltration (ICI), immune checkpoint expression, and stromal cell activities. Notably, YTHDF1 and YTHDF2 promote the recruitment of tumor-infiltrating lymphocytes (TILs), enhancing immune surveillance while simultaneously suppressing PD-L1 expression to facilitate immune cell-mediated tumor destruction, thus establishing an inflammatory tumor milieu (139). Through GO and KEGG enrichment analyses, Zhu et al. identified 15 differentially expressed genes (DEGs) enriched in immune-related biological processes. This demonstrates that m6A modification patterns impact the TME by regulating these genes (140). However, since this research was conducted *in vitro*, it might not fully replicate the tumor’s surrounding microenvironment, potentially not accurately reflecting *in vivo* observations.

Furthermore, Du et al. used m6A-Seq and RNA-Seq analysis to discover that SUMOylation of METTL3 leads to significant changes in gene expression profiles. These changes could influence intercellular signaling in the TME, thereby promoting tumor progression. Additional studies have shown that METTL3 promotes the m6A modification and stabilization of miR-196a, which targets the 3’UTR of growth arrest-specific 7 (GAS7) to suppress its expression. Diminished GAS7 levels are associated with reduced ICI into tumors, facilitating immune evasion and promoting NSCLC progression (115). guanylate-binding protein 4 (GBP4) expression in NSCLC is positively correlated with immune-regulatory chemokines, including CXCL9, CXCL10, and CCL5, which are pivotal for the recruitment of CD8+ T cells, macrophages, and antigen-presenting cells (141). Meanwhile, interactions between IGF2BPs and circNDUFB2 promote circNDUFB2 degradation, helping tumor cells evade immune detection (142).

Emerging evidence highlights the role of cancer-associated fibroblasts (CAFs) in modulating the NSCLC microenvironment. CAFs can transfer METTL3 to tumor cells via secreted factors and exosomes, elevating intracellular m6A levels and altering gene expression profiles such as SLC7A5 to support tumor growth (116). Additionally, CAF-derived VEGFA upregulates METTL3 in NSCLC cells and activates the AKT/NF-κB pathway via RAC3, further promoting m6A methylation and enhancing tumor migration and invasion (143). Gu et al. demonstrated that HNRNPC expression fosters an immunosuppressive TME by regulating PD-L1 levels and modulating ICI. Conversely, HNRNPC knockdown increases CD8+ T cell infiltration and alters stromal composition, potentially augmenting anti-tumor immunity *in vivo (* 138).

#### 5.1.2 Angiogenesis

m6A RNA modification acts as a central regulator in the angiogenesis process of NSCLC by modulating the expression and stability of several key molecules. TRPM7 enhances the expression of angiogenesis factors, such as VEGFA, by activating specific pathways, thereby promoting tumor angiogenesis (144, 145). Recent findings also show that m6A modification stabilizes TRPM7 mRNA through IGF2BP2-mediated mechanisms, upregulating TRPM7 expression in NSCLC (42). lncRNA DGUOK-AS1, found to be overexpressed in NSCLC, binds IGF2BP2 to enhance TRPM7 mRNA stability, subsequently promoting VEGFA expression and facilitating angiogenesis (146). Moreover, METTL3-mediated stabilization of FMOD mRNA via increased m6A modification levels leads to elevated expression of pro-angiogenic factors, further driving tumor vascularization (147).

#### 5.1.3 Glycolysis

Mounting evidence supports the critical role of m6A methylation in regulating metabolic reprogramming in NSCLC. It prominently influences the Warburg effect by precisely modulating the expression of glycolysis-related genes. Gu et al., through gene set enrichment analysis (GSEA), revealed that m6A methylation alters the metabolic state of NSCLC cells by regulating glycolytic gene expression. Elevated m6A levels activate glycolysis, providing energy and biosynthetic intermediates essential for rapid tumor proliferation (148). Of particular importance is phosphofructokinase, liver type (PFKL), the second rate-limiting enzyme in glycolysis, which catalyzes the phosphorylation of fructose-6-phosphate to fructose-1,6-bisphosphate, a key step in the glycolytic pathway. Increased PFKL activity directly enhances glycolysis, enabling cells to convert glucose to lactate more efficiently while generating ATP (149, 150).

circDHTKD1 binds with IGF2BP2, enhancing IGF2BP2’s binding capability to PFKL mRNA, thereby stabilizing the mRNA. This regulatory mechanism increases PFKL protein expression, promoting glycolysis in NSCLC cells (118).ABHD11-AS1 is primarily located in the nucleus of NSCLC cells, suggesting that it may influence glycolysis through transcriptional regulation (151).

#### 5.1.4 Ferroptosis

Ferroptosis is an iron- and reactive oxygen species (ROS)-dependent form of regulated cell death, closely associated with cellular redox homeostasis (152). Recent studies have identified multiple high-confidence m6A modification sites on the mRNA of Ferroptosis Suppressor Protein 1 (FSP1), which modulate the stability and translational efficiency of FSP1 transcripts, consequently influencing protein abundance. In NSCLC, enhanced m6A methylation may reduce FSP1 expression, thereby facilitating ferroptosis induction (132). Furthermore, m6A modifications impact the expression of genes involved in iron metabolism and ROS production, modulating iron uptake, storage, and utilization, as well as ROS generation and detoxification, thus affecting intracellular iron levels and ferroptosis dynamics (153). KIAA1429, a key component of the m6A methyltransferase complex, plays a crucial role across various malignancies (154, 155). Wu et al. demonstrated that silencing KIAA1429 via siRNA augments the sensitivity of NSCLC cells to the ferroptosis inducer erastin, characterized by increased intracellular Fe^2+^ and ROS accumulation, reduced levels of GSH and MDA, elevated PTGS2 expression, and decreased GPX4 and FTH1 protein levels (156).

Similarly, ALKBH5 overexpression in NSCLC cells leads to diminished GSH levels, accompanied by increased lipid ROS, malondialdehyde (MDA), and iron accumulation—hallmarks of ferroptosis (157). SLC3A2, the heavy chain subunit of the system Xc^-^ cystine/glutamate antiporter, is another critical regulator of ferroptosis. Elevated SLC3A2 expression enhances GSH biosynthesis, thereby suppressing ferroptosis by promoting cellular antioxidant capacity (158). Chen et al. demonstrated through cellular experiments that altering METTL14 expression in LC cells directly affects m6A modification levels and protein expression of SLC3A2. Knockdown of METTL14 increases SLC3A2 expression, reducing sensitivity to ferroptosis inducers like Erastin, whereas METTL14 overexpression decreases SLC3A2 expression, increasing sensitivity to ferroptosis (122).

While individual roles of m6A regulators and ferroptosis-related genes in NSCLC have been extensively explored, their co-expression dynamics and regulatory interdependencies remain insufficiently characterized. Recent evidence indicates that expression levels of certain m6A “writers”, “erasers”, and “readers” may exhibit coordinated or antagonistic patterns with ferroptosis mediators such as SLC7A11, FSP1, and GPX4 (134). For example, ALKBH5 downregulation correlates with increased SLC7A11 expression and ferroptosis resistance (159), while IGF2BP3 stabilizes FSP1 transcripts, contributing to ferroptosis suppression (160).

These findings suggest a broader regulatory axis in which m6A modifications modulate ferroptosis sensitivity through altering the stability or translation of genes involved in redox balance and iron metabolism (161). Future mechanistic studies integrating multi-omics data are warranted to elucidate whether these interactions represent compensatory networks or convergent regulatory circuits that could be therapeutically targeted in NSCLC.

#### 5.1.5 lncRNAs

A complex regulatory network between m6A modifications and lncRNAs critically influences NSCLC development by modulating signaling cascades related to proliferation and apoptosis. Li et al. found that HNRNPA2B1 regulates the function of lncRNA MEG3 by stabilizing it through m6A-dependent mechanisms. lncRNA MEG3 acts as a molecular sponge for miR-21-5p, suppressing its activity. Since miR-21-5p targets PTEN and activates the PI3K/AKT pathway to promote cell proliferation and survival, MEG3-mediated sequestration of miR-21-5p restores PTEN expression and thereby inhibits oncogenic signaling, restraining tumorigenesis (136). CDC6, a pivotal cell cycle regulator, is implicated in the initiation and progression of several cancers (162). In NSCLC, the lncRNA LCAT1 stabilizes IGF2BP2, which, in turn, regulates CDC6 expression via m6A modification, forming a signaling axis that promotes tumor cell proliferation. Activation of this axis fosters NSCLC cell growth, whereas its inhibition impairs tumor proliferation (163).

Interestingly, research has shown that ALKBH5 enhances the stability of CALML3-AS1 by removing its m6A modification, promoting NSCLC cell proliferation. Conversely, YTHDC2 recognizes the m6A modification on CALML3-AS1, facilitating its degradation and suppressing NSCLC cell proliferation. CALML3-AS1 recruits EZH2 to inhibit BTNL9 expression, where high BTNL9 expression suppresses NSCLC cell proliferation and migration, potentially through the induction of apoptosis (129). *In vitro* studies have shown that RMRP overexpression significantly enhances the proliferation of NSCLC cell lines, such as A549 and H1299. Conversely, RMRP knockdown markedly suppresses cell viability, as evidenced by CCK-8 and colony formation assays. RMRP affects apoptosis in NSCLC cells by regulating the TGFBR1/SMAD2/SMAD3 pathway. Knockdown of RMRP increases the BAX/Bcl-2 ratio, suggesting that RMRP may influence apoptosis through regulating apoptosis-related gene expression (164).

Liang et al. found that lncRNA KCTD21-AS1 is upregulated in NSCLC tissues. The m6A modification of KCTD21-AS1 suppresses miR-519d-5p activity, leading to increased expression of CD47 and TIPRL. Elevated CD47 inhibits macrophage phagocytosis of tumor cells, while higher TIPRL levels enhance tumor cell proliferation and survival. Conversely, as an inhibitor of PP2A, TIPRL modulates apoptosis-related signaling pathways to suppress apoptosis, thereby promoting tumor cell survival and proliferation (165). Further investigations revealed that METTL14, a core methyltransferase, downregulates LINC02747 expression via m6A modification, thereby inhibiting the PI3K/AKT signaling cascade, reducing cell proliferation and migration, and promoting apoptosis. KDM5B, through H3K4me3 demethylation, represses METTL14 transcription, leading to upregulation of LINC02747 and enhancing NSCLC malignancy (121).

### 5.2 Role of m6A in LC invasion and metastasis

#### 5.2.1 Angiogenesis

m6A modifications play a critical role in regulating angiogenesis in NSCLC, where interactions between cancer-associated fibroblasts (CAFs) and m6A modifications are central in shaping the expression and stability of angiogenic factors such as VEGFA, crucial for tumor vasculature formation. Notably, different m6A-related proteins might distinctly influence angiogenesis by remodeling the TME and modifying the extracellular matrix composition. Specifically, the interaction network between m6A-related proteins and CAFs establishes a sophisticated regulatory network that coordinates tumor angiogenesis through multiple signaling pathways. Existing studies have identified the VEGFA signaling pathway as the central pathway for angiogenesis (166). In NSCLC, CAFs affect m6A modifications on VEGFA mRNA, enhancing its stability and translational efficiency, which boosts VEGFA secretion. RAC3, a downstream target gene of METTL3, shows significantly increased m6A modification and protein expression after CAF treatment.

RAC3 may further enhance the migratory and invasive abilities of NSCLC cells by activating the AKT/NF-κB signaling pathway. Aside from RAC3, other angiogenesis-associated genes whose m6A modification levels are altered with CAF treatment are MMP9 (Matrix Metallopeptidase 9) and Twist1. m6A modification of these genes might regulate their expression, which, in turn, controls angiogenesis as well as the invasion and metastasis of NSCLC (143). On the other end, immunofluorescence (IF) examination of increased CD31 fluorescence density, an endothelial cell marker, in HNRNPC-knockdown tumor tissues hints at angiogenesis inhibition. Knocking down HNRNPC is associated with upregulation of collagen I expression, a significant extracellular matrix (ECM) composition. Such a change might suppress angiogenesis, inhibit cancer progression, and reorganize the TME to lean towards anti-tumor responsiveness (138).

#### 5.2.2 Glycolysis

m6A modifications have a significant effect on metabolic reprogramming in NSCLC through regulation of the stability and activity of crucial lncRNAs, as well as of key metabolic regulators such as c-Myc. This regulatory network plays a crucial role in the control of aerobic glycolysis, a characteristic feature of the Warburg effect. ABHD11-AS1 binds to EZH2, a PRC2 complex member, and recruits it to the KLF4 promoter, repressing KLF4 transcription (167). Because KLF4 is a tumor suppressor that can suppress glycolysis, its repression by ABHD11-AS1 facilitates glycolytic metabolism as well as cancer progression of NSCLC (168). Besides, m6A modification promotes the stability of DLGAP1-AS2, enabling its function as a stimulant of aerobic glycolysis. High expression levels of DLGAP1-AS2 are associated with elevated levels of glucose uptake, lactate output, ATP delivery, as well as NSCLC cell growth. Concomitant with it, m6A-modified c-Myc mRNA is stabilized by YTHDF1-dependent mechanisms, resulting in upregulation of the protein level of c-Myc. As a master transcription factor of glycolysis, upregulation of c-Myc expression promotes aerobic glycolysis, powering rapid growth of tumor cells (169).

#### 5.2.3 Ferroptosis

m6A demethylase ALKBH5 plays a dual role in the regulation of ferroptosis in NSCLC through regulation of the expression level of the key subunit of system Xc^-^, which is SLC7A11. It impacts the oxidative stress equilibrium as well as iron homeostasis, hence having a substantial regulatory function in cancer growth. Huang et al. established through studies that ALKBH5 decreases the levels of SLC7A11 by erasing m6A modifications from its mRNA (128). SLC7A11 is a subunit of system Xc^-^ that imports cystine to synthesize glutathione (GSH), enhancing the cell’s antioxidant capacity (170). Upregulation of ALKBH5 promotes ferroptosis in NSCLC cells by decreasing SLC7A11 expression, thereby inhibiting tumor invasion and metastasis. However, high expression of ALKBH5 can also inhibit ferroptosis, promoting tumor progression.

Overexpression of SLC7A11 can partially counteract the ferroptosis-promoting effect of ALKBH5 overexpression, as evidenced by elevated GSH levels, reduced lipid ROS, MDA, and iron ion content, enhanced cell proliferation, migration, invasion capabilities, and reduced apoptosis rates (128). Recent studies have identified IGF2BP3 as an m6A reader protein that critically regulates ferroptosis by stabilizing the mRNA of anti-ferroptotic genes, thereby inhibiting ferroptotic cell death and enhancing tumor cell survival and invasion capabilities (134).

#### 5.2.4 lncRNAs

m6A modifications also play a pivotal role in regulating the expression and function of numerous lncRNAs, which collectively promote NSCLC invasion and metastasis through mechanisms such as competing endogenous RNA (ceRNA) networks, epigenetic silencing, and TME remodeling. Research indicates that CALML3-AS1, regulated via m6A modifications by ALKBH5 and YTHDC2, facilitates NSCLC cell proliferation, migration, invasion, and epithelial-mesenchymal transition (EMT), while its silencing suppresses these malignant behaviors. Additionally, CALML3-AS1 recruits EZH2 to epigenetically suppress BTNL9 expression, facilitating NSCLC invasion and metastasis. Overexpression of BTNL9 inhibits the malignancy of NSCLC cells, and its knockdown partially counteracts the inhibitory effects of CALML3-AS1 knockdown (129). Functional experiments by Song et al. revealed that m6A modifications regulate FEZF1-AS1 expression, allowing it to competitively bind miR-516b-5p, which upregulates ITGA11 expression, thereby promoting NSCLC cell invasion and metastasis (130).

YAP is a key effector in the Hippo pathway, whose aberrant activation is associated with various cancers (171). *In vivo* and *in vitro* studies demonstrate that METTL3 enhances the m6A methylation of MALAT1, increasing its transcript stability and upregulating YAP expression through the MALAT1–miR-1914-3p–YAP axis, thereby promoting NSCLC invasion and metastasis (172). Additionally, RBM15, functioning as an m6A methyltransferase, stabilizes CBR3-AS1 via m6A modifications. This stabilization activates the miR-409-3p/CXCL1 axis to recruit myeloid-derived suppressor cells (MDSCs), suppress T cell responses, and contribute to radioresistance in NSCLC. This process not only reduces the efficacy of radiotherapy but may also enhance tumor invasion and metastasis by modulating the immune microenvironment (125).

### 5.3 Subtype-specific m6A regulation in LUAD and LUSC

NSCLC is primarily divided into two major subtypes: lung adenocarcinoma (LUAD) and lung squamous cell carcinoma (LUSC). Although both subtypes fall under the umbrella of NSCLC, they demonstrate distinct molecular mechanisms and biological characteristics (173). These distinctions manifest in gene expression profiles, metabolic reprogramming, and immune response regulation. Studies have demonstrated that LUAD cells frequently exhibit enhanced metabolic reprogramming, particularly mediated by m6A modifications affecting glycolysis and ferroptosis pathways, thereby facilitating rapid proliferation and metastasis (174). Furthermore, LUAD is frequently characterized by enhanced immune evasion, closely associated with m6A-mediated regulation of immune checkpoint molecules such as PD-L1 (113). In contrast, LUSC predominantly depends on an immunosuppressive tumor microenvironment to support tumor progression, especially through immune evasion (175). In LUSC, m6A modifications upregulate the expression of immune checkpoint molecules, including PD-L1, leading to T cell suppression and promoting immune evasion (176). These observations suggest mechanistic differences in m6A modification between LUAD and LUSC: LUAD primarily promotes tumor progression via metabolic reprogramming, whereas LUSC emphasizes immune evasion strategies.

The role of m6A modification in LUAD and LUSC cell carcinoma extends beyond metabolic and immune regulation, encompassing the modulation of distinct m6A regulatory factors (177). In LUAD, m6A modification enhances the expression of several key genes, including the stabilization of TRPM7 mRNA by IGF2BP2, which subsequently upregulates VEGFA expression and promotes angiogenesis (13, 146). Moreover, m6A plays a pivotal role in glycolysis, particularly through stabilization of PFKL mRNA, thereby sustaining the energy supply and proliferation of LUAD cells (178). In LUSC, m6A modification facilitates immune evasion by upregulating immune checkpoint molecules such as PD-L1, thereby impairing immune cell function and promoting tumor progression (114). Furthermore, in LUSC, m6A modification promotes the secretion of VEGFA and other cytokines by cancer-associated fibroblasts (CAFs), thereby remodeling the tumor microenvironment and enhancing angiogenesis and metastasis (179). Additionally, LUAD and LUSC exhibit subtype-specific differences in the m6A modification of particular long non-coding RNAs (lncRNAs). For instance, in LUAD, m6A modification enhances the stability of MALAT1, thereby promoting tumor metastasis (133); in contrast, in LUSC, m6A facilitates immune evasion by modulating the expression of immune-related genes (180).

Interestingly, an increasing number of recent studies have utilized publicly available datasets, particularly the TCGA and GEO databases, to reveal substantial differences in the expression and function of m6A regulators among NSCLC subtypes. Analysis of the TCGA database has shown that several key m6A regulatory factors, such as YTHDF2, METTL3, and FTO, exhibit significant expression differences in NSCLC and are closely associated with clinicopathological features and patient prognosis (181). For example, Zhao et al. analyzed the TCGA lung adenocarcinoma dataset and found that YTHDF2 is highly expressed in LUAD and associated with better overall survival, possibly by inhibiting the FAM83D–TGFβ1–pSMAD2/3 pathway to suppress metastasis (182); In contrast, Yang et al. found that increased METTL3 expression promotes miR-196a expression and tumor progression via m6A modification (115).Additionally, Li et al., through a study of 1,057 NSCLC cases from the TCGA database, found that copy number variations in m6A regulators, such as deletions in FTO and YTHDC2, are associated with poor prognosis in NSCLC patients (183). m6A modification also broadly influences the expression levels of non-coding RNAs (ncRNAs). For instance, METTL3-mediated m6A modification stabilizes LINC01006, forming a c-MYC/METTL3/LINC01006 positive feedback loop that promotes NSCLC cell migration and proliferation (184). Another study developed a risk score model based on 18 m6A-related ncRNAs using TCGA data, revealing that high-risk patients have worse prognosis, and the risk score is significantly associated with tumor size, lymph node metastasis, clinical stage, sex, and NSCLC subtype (185).

In summary, m6A modification exhibits distinct subtype-specific roles in LUAD and LUSC. In LUAD, m6A primarily facilitates tumor progression via metabolic reprogramming and angiogenesis, whereas in LUSC, it predominantly acts through immune evasion mechanisms. These findings provide a theoretical basis and identify potential therapeutic targets for the precision treatment of NSCLC.

## 6 Clinical value of m6A modifications

### 6.1 Potential as diagnostic biomarkers

The application of m6A modification in NSCLC diagnosis and treatment, particularly via liquid biopsy, is receiving growing attention. Liquid biopsy, a minimally invasive technique analyzing blood samples for circulating tumor DNA (ctDNA), circulating tumor cells (CTCs), and exosomes, offers advantages such as repeatability, lower risk, and faster turnaround (186). Utilizing m6A RNA immunoprecipitation (MeRIP) combined with real-time quantitative RT-PCR, Zhang et al. successfully detected m6A modifications on CD47 mRNA from liquid biopsy samples, highlighting a novel approach for monitoring tumor-derived RNA (187). Further studies have shown that exosomes from cisplatin-resistant (CIS-R) tumors deliver miR-4443 to cisplatin-sensitive (CIS-S) cells, conferring drug resistance, suggesting miR-4443 as a potential biomarker for cisplatin response in NSCLC (188). Similarly, elevated levels of serum m6A-modified miR-17-5p have been detected in early-stage NSCLC patients, suggesting its potential as a non-invasive early diagnostic marker (189, 190).

### 6.2 Therapeutic strategies and overcoming drug resistance

Therapeutic strategies targeting the m6A modification pathway show great potential in NSCLC treatment by regulating the cell cycle, drug sensitivity, ferroptosis, and immune response, offering new intervention targets to overcome chemotherapy resistance and enhance immunotherapy effectiveness. Yang et al. discovered that the therapeutic role of m6A modification in NSCLC is predominantly through influencing cell cycle progression via the regulation of key gene expression, such as CDK2AP2, in lung tissue of smokers. Inhibiting crucial factors in the m6A pathway, like METTL3 and HIF-1α, can offer novel treatment strategies for NSCLC, particularly for smoking-related patients (191).Research also indicates that METTL3 regulates the expression of resistance-related genes via m6A modification, such as enhancing SOX2 stability to promote cisplatin resistance. Therefore, inhibiting methyltransferase activity like METTL3 or activating demethylases like FTO or ALKBH5 can reduce m6A modification levels, thereby inhibiting tumor progression and resistance (192).

Cheng et al. revealed that smoking-induced modulation of TAMs is mediated through circRNA circEML4. By altering ALKBH5 activity or blocking its interaction with circEML4, researchers reduced SOCS2 regulation via m6A modification, thereby inhibiting JAK-STAT pathway activation and slowing NSCLC progression (131). Additionally, the lncRNA LINC02418 enhances Trim21-mediated ubiquitination of PD-L1, reducing its expression and increasing T cell-mediated cytotoxicity. Clinical analyses demonstrated that METTL3 regulates LINC02418 stability via m6A methylation, with YTHDF2 facilitating its degradation. Inhibiting METTL3 thus elevates LINC02418 expression, decreases PD-L1 levels, and may enhance the efficacy of immunotherapy (114).Moreover, m6A modifications can modulate ferroptosis-related gene expression, suggesting ferroptosis promotion as a novel therapeutic avenue in LC. Inhibition of the m6A demethylase FTO elevates m6A methylation, thereby inducing ferroptosis and suppressing LC cell proliferation and migration (122).

KIAA1429, the largest subunit of the m6A methyltransferase complex, acts as a scaffold linking catalytic core components to RNA substrates, dictating site-specific m6A deposition (193). Lin et al. demonstrated that KIAA1429 regulates MAP3K2 expression via m6A methylation, activating the JNK/MAPK pathway and promoting gefitinib resistance; silencing KIAA1429 restores gefitinib sensitivity in LUAD cells (123). VIRMA, another essential MTC component, mediates m6A deposition on RNA and exhibits oncogenic roles in NSCLC. Inhibiting VIRMA reduces m6A modification on DAPK3 mRNA, thereby enhancing DAPK3 expression and restoring its tumor-suppressive functions (124). Trans-3,5,4’-trimethoxystilbene (TMS), a natural compound with potent anti-tumor properties, has been shown to inhibit m6A methylation of circPACRGL. This suppression impairs the function of circPACRGL in both NSCLC cells and exosomes, limiting tumor progression and reducing M2 macrophage polarization (194, 195). These findings underscore m6A methylation as a promising therapeutic target in NSCLC.

### 6.3 Prognostic assessment

Multiple studies have demonstrated that the expression levels of m6A-related regulatory proteins, are closely associated with the prognosis of NSCLC patients (196). METTL3 has been shown to repress RIG-I expression through m6A methylation, thereby inhibiting the activation of the RIG-I-MAVS innate immune pathway. Since activation of this pathway suppresses tumor growth and stimulates anti-tumor immunity, its inhibition may allow NSCLC cells to evade immune surveillance, facilitating tumor progression and metastasis. Consequently, elevated METTL3 and reduced RIG-I expression levels may serve as potential biomarkers of poor prognosis (197).

Further investigations into how m6A modification promotes stem-like characteristics in NSCLC identified a positive feedback loop between the lncRNA SOX2OT and GLI1. Inhibition of either GLI1 or METTL3 significantly impairs the tumorigenic potential of LC cells. In nude mouse models, administration of the GLI1 inhibitor GANT58 and the METTL3 inhibitor SAH, individually or in combination, markedly delayed tumor growth, with the combined treatment showing superior efficacy. These findings suggest that pharmacological targeting of m6A methylation and its associated pathways holds promise for improving LC prognosis (198).

Similarly, Zhou and colleagues developed a Writer-Score system based on differentially expressed genes (DEGs) to quantify RNA modification patterns and predict clinical outcomes. Incorporating multiple RNA modification writers, including m6A enzymes, the study demonstrated that patients with low Writer-Scores exhibited better prognoses, while those with high scores had worse outcomes. Elevated expression of m6A regulators in the high Writer-Score group further supports the association between m6A modification and unfavorable clinical progression (199).

### 6.4 Comparative analysis of m6A and DNA methylation detection technologies

Accurate and reproducible detection technologies are fundamental to the clinical translation of epigenetic biomarkers. DNA methylation detection has been extensively optimized, with bisulfite sequencing, pyrosequencing, and methylation-specific PCR enabling single-CpG resolution and broad clinical applicability (200).

By contrast, m6A RNA methylation detection poses unique technical challenges due to the lack of chemical reactivity akin to bisulfite conversion. Early antibody-based techniques such as MeRIP-seq offered transcriptome-wide mapping but lacked base-level precision (201). Technological innovations such as miCLIP, m6A-REF-seq, DART-seq, and SELECT have since achieved single-nucleotide resolution and increased sensitivity. However, these methods still face hurdles in standardization, throughput, and antibody specificity (84).

In terms of clinical feasibility, DNA methylation assays are more cost-effective and robust across laboratories. m6A detection, though promising, remains largely confined to research settings. Importantly, its dynamic and reversible nature offers a potential advantage for monitoring real-time treatment response or tumor evolution (202). A synergistic diagnostic strategy combining DNA and RNA methylation profiling may enhance the sensitivity and specificity of NSCLC early detection and patient stratification.

## 7 Conclusions and future perspectives

### 7.1 Summary of key findings

**Figure 3:**
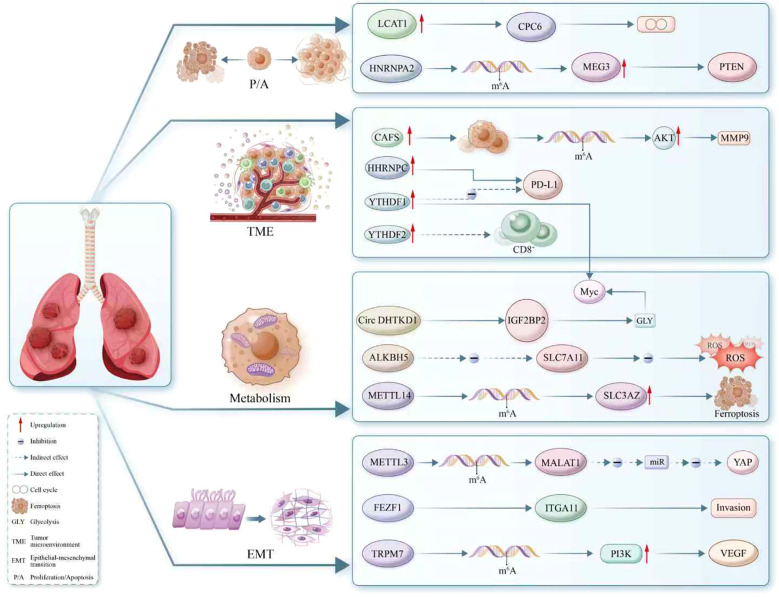
The regulatory landscape of m6A modification in RNA metabolism within non-small cell lung cancer (NSCLC). This schematic delineates the complex interplay between m6A-modified RNA and key oncogenic processes, including proliferation, apoptosis, epithelial-mesenchymal transition (EMT), tumor microenvironment modulation, glycolysis, and ferroptosis. Enzymes such as METTL3 and METTL14 serve as ‘writers’ of the m6A mark, while ‘erasers’ like ALKBH5 reverse the modification, highlighting the dynamic regulation of RNA fate. The role of ‘readers’ such as YTHDF1 and IGF2BP2 is showcased, with specific emphasis on their ability to direct RNA towards distinct cellular pathways. Illustrated interactions involve oncogenes and tumor suppressors like PTEN, MYC, and PD-L1, and the m6A mark’s influence on cellular components like circRNAs, lncRNAs (e.g., MALAT1, MEG3), and miRNAs. Furthermore, the diagram reveals m6A’s impact on regulators of immune cell function (CD8+), metabolic enzymes (SLC7A11), and components of the extracellular matrix (MMP9), underscoring its pivotal role in NSCLC biology. This comprehensive depiction emphasizes m6A as a crucial epitranscriptomic mediator orchestrating diverse and vital cellular processes.

RNA m6A modification serves as a central mechanism of epitranscriptomic regulation, orchestrating RNA metabolism through a finely controlled enzymatic network (**Figure 3**). Recent findings have underscored its critical role in maintaining pulmonary microenvironmental homeostasis and its multifaceted regulatory influence, particularly in the context of NSCLC. Experimental evidence indicates that m6A modification significantly influences tumor biology through multiple mechanisms, including restructuring the immune microenvironment, reprogramming metabolic networks, and regulating iron homeostasis. As detection technologies have evolved, from early immunoprecipitation-based omics methods to novel single-base resolution techniques, researchers can now analyze m6A modification patterns with varying precision levels.

The emergence of innovative methods like metabolic labeling and direct detection offers unprecedented technological support for elucidating the functional mechanisms of m6A.In translational medicine, the expression patterns of m6A-related regulatory factors show significant correlation with patient prognosis. Notably, methyltransferases like METTL3 offer new insights for optimizing immunotherapy strategies through their regulatory roles in immune response and microenvironmental remodeling. Additionally, metabolic regulation and ferroptosis pathway intervention based on m6A modification show potential in overcoming treatment resistance. These findings not only deepen the understanding of RNA epigenetic modifications in tumorigenesis but also offer new molecular targets for developing precision therapeutic strategies.

### 7.2 Interactions with Other RNA modifications

In NSCLC research, although the m6A modification has been extensively studied due to its widespread distribution and dynamic reversibility, other RNA modifications also play significant roles in the initiation and progression of lung cancer (**Table 3**). These modifications may evolve into crucial regulatory factors beyond m6A in the future. Among these, 1-methyladenosine (m1A) modification is predominantly found in the 5′UTRs of tRNA, rRNA, and certain mRNAs (206). It is catalyzed by methyltransferases such as TRMT6/TRMT61A, which can alter the local structure of RNA, thereby influencing ribosome recognition and translation efficiency (207). Studies have demonstrated that aberrant m1A modification can lead to metabolic reprogramming in cancer cells and increased resistance to chemotherapy (208). While the precise mechanisms underlying m1A modification in NSCLC remain unclear, its potential impact warrants further investigation. Additionally, N4-acetylcytidine (ac4C) is a rare modification catalyzed by NAT10, primarily enhancing mRNA stability and translation efficiency (209). It has been shown to promote oncogenesis in other cancers by regulating inflammatory factors, the cell cycle, and metabolic pathways (210). Although research on lung cancer remains limited, the natural expression of NAT10 in lung tissue, along with its association with inflammation and tumors, suggests its potential role in regulating the NSCLC microenvironment (211).

**Table 3:** Key RNA modifications and their functional roles in NSCLC.

Modification Type	Main Modified Base	Writers	Erasers	Readers	Functional Role in NSCLC	Ref
m6A	Adenosine (A), N6 position	METTL3, METTL14, WTAP, VIRMA, RBM15, ZC3H13, METTL16	FTO, ALKBH5	YTHDF1/2/3, YTHDC1/2, IGF2BP1/2/3, eIF3	Regulates NSCLC cell proliferation, apoptosis, ferroptosis, glycolysis, immune evasion, and tumor microenvironment remodeling; interacts extensively with lncRNA	(164)
m1A	Adenosine (A), N1 position	TRMT6, TRMT61A, TRMT10C	ALKBH1, ALKBH3	—	Regulates mRNA secondary structure stability and translation efficiency affecting NSCLC development	(196)
ac4C	Cytidine (C), C4 acetylation	NAT10	—	—	Enhances mRNA stability and translation efficiency; may be involved in stress response and metabolic regulation in lung cancer	(203)
m5C	Cytidine (C), C5 methylation	NSUN2, DNMT2, NSUN6	TET family	ALYREF, YBX1, RAD52	Regulates mRNA stability, nuclear export, and translation; high expression associated with NSCLC metastasis and drug resistance	(204)
m7G	Guanosine (G), G7 methylation	METTL1, WDR4	—	eIF4E	Affects translation initiation and mRNA lifespan; promotes tumor progression in NSCLC by enhancing protein synthesis and metabolic activity in cancer cells	(205)

m6A, N6-methyladenosine; m1A, N1-methyladenosine; ac4C, N4-acetylcytidine; m5C, 5-methylcytidine; m7G, 7-methylguanosine; NSCLC, Non-small cell lung cancer; WTAP, Wilms tumor 1 associated protein; VIRMA, Vir-like m6A methyltransferase associated; RBM15, RNA binding motif protein 15; YTHDF/DC, YT521-B homology domain family/containing; IGF2BP, Insulin-like growth factor 2 mRNA-binding protein; eIF, Eukaryotic initiation factor; TRMT, tRNA methyltransferase; NAT10, N-acetyltransferase 10; NSUN, NOP2/Sun RNA methyltransferase; DNMT2, DNA methyltransferase 2; TET, Ten-eleven translocation enzyme family; ALYREF, Aly/REF export factor; YBX1, Y-box binding protein 1; RAD52, RAD52 homolog; WDR4, WD repeat domain 4.

5-methylcytosine (m5C) modification has been extensively reported as abnormally expressed in NSCLC, especially in the methyltransferase NSUN2 (212). This modification enhances mRNA nuclear export, stability, and translation efficiency, while also promoting tumor cell migration and invasion through the regulation of oncogenes such as CDK and ZEB1 (213). Recent studies have also shown that m5C modification is associated with radiotherapy resistance, suggesting its potential as an intervention target for combination therapies (214). Another key modification, N7-methylguanosine (m7G), is predominantly found in the mRNA cap structure and tRNA (215). The m7G modification, regulated by the METTL1/WDR4 complex, enhances mRNA translation efficiency (216). Recent studies have shown that it promotes cell cycle progression and glycolysis in lung cancer through the regulation of the Myc pathway and PI3K/AKT signaling (217).

In summary, although these RNA modifications each possess distinct characteristics, they all contribute to the progression of NSCLC by regulating RNA fate. In the future, multi-omics analysis can be employed to jointly explore the interaction networks among various RNA modifications, investigate their synergistic regulatory mechanisms, and aim to construct a more systematic epitranscriptomic map of lung cancer, which will lead to improved outcomes for NSCLC patients.

### 7.3 Challenges and strategies for clinical translation

Even though m6A modification holds promise for NSCLC studies, its clinical application is confronted with various challenges. Firstly, the unavailability of standard methods of detecting m6A modifications poses constraints for its clinical application, as existing methods are complicated and expensive (218). Secondly, poor awareness of clinicians impedes the utilization of m6A modifications for practical diagnosis as well as treatment. For its improvement, multicenter collaborative studies are suggested to promote the development of systematic methods for detecting m6A. Second, increased training of clinicians can improve their understanding of m6A modifications. Third, increased funding from government as well as research organizations is imperative to promote the clinical application of m6A modifications for NSCLC. All these actions are expected to surpass existing hurdles to accelerate the application of m6A modifications for NSCLC diagnosis as well as treatment.
